# Comparisons in Postoperative Endoscopic Findings and Postoperative Weight Change Between Delta‐Shaped Anastomosis and Circular‐Stapled Anastomosis in Laparoscopy‐Assisted Distal Gastrectomy With B‐I Reconstruction

**DOI:** 10.1111/ases.70023

**Published:** 2025-01-23

**Authors:** Shuichiro Oya, Shinichi Sakuramoto, Yosuke Morimoto, Kazuaki Matsui, Keiji Nishibeppu, Gen Ebara, Shohei Fujita, Shiro Fujihata, Seigi Lee, Yutaka Miyawaki, Hirofumi Sugita, Hiroshi Sato, Keishi Yamashita

**Affiliations:** ^1^ Department of Gastroenterological Surgery Saitama Medical University International Medical Center Saitama Japan; ^2^ Department of Gastrointestinal Surgery The University of Tokyo Tokyo Japan; ^3^ Division of Advanced Surgical Oncology, Research and Development Center for New Medical Frontiers Kitasato University School of Medicine Sagamihara Japan

**Keywords:** Billroth I, gastrectomy, gastritis, laparoscopic surgery, surgical anastomosis

## Abstract

**Background:**

Laparoscopy‐assisted distal gastrectomy (LADG) with Billroth I (B‐I) reconstruction is frequently performed for gastric cancer. However, the difference between the circular stapler technique (CS) and delta‐shaped anastomosis (DA) remains unclear, especially regarding the postoperative endoscopic physiological findings.

**Methods:**

Three hundred and one patients including 150 CS patients and 151 DA patients during LADG with B‐I reconstruction between 2013 and 2019 at Saitama Medical University International Medical Center were chosen as study subjects. Postoperative endoscopic findings (1‐year post‐surgery) in the remnant stomach were evaluated according to the residue, gastritis, and bile‐reflux classification, and the first‐year postoperative weight changes were also recorded.

**Results:**

The incidences of Grade 2 or higher remnant gastritis, bile reflux, and postoperative exacerbated reflux esophagitis were significantly higher in the DA group, while the amount of residual food was higher in the CS group. Multivariate analysis also revealed the higher risks of Grade 2 or higher gastritis and the postoperative existence or exacerbation of erosive reflux esophagitis in the DA group (OR [95% CI] was 2.737 [1.566–4.783], 3.533 [1.101–11.34], and 3.749 [1.021–13.76], respectively). However, none of these endoscopic differences but the broader extent of gastritis was the only endoscopic factor associated with severe postoperative weight loss.

**Conclusion:**

There was a trend toward more exacerbation of residual gastritis and reflux esophagitis with the DA technique and more food remnants with the CS technique. Although the difference in the anastomotic technique did not directly result in weight loss, attention should be paid to prevent extensive residual gastritis.

## 1 Introduction

Laparoscopy‐assisted distal gastrectomy (LADG) is becoming a common and standard surgical intervention for gastric cancer in Japan. A study from the Japanese Clinical Oncology Group for gastric cancer revealed the non‐inferiority of LADG for oncological and postoperative outcomes for stage IA and IB gastric cancer patients when compared to open gastrectomy [1]. Non‐inferiority of LADG for disease‐free survival at 3 years after surgery for locally advanced cases was also recently shown in the CLASS‐01 randomized clinical trial [2].

The developments of novel surgical techniques, energy devices, and automatic suture staplers have made surgical procedures more accessible and accelerated the prevalence of LADG. The first laparoscopy‐assisted Billroth I gastrectomy (B‐I) was performed by Kitano et al. and was published in 1994 [3]. LADG was also established for other prevalent open surgery reconstructive techniques, such as Billroth II (B‐II) and Roux‐en‐Y (R‐Y). These reconstructive techniques were previously performed with minimal laparotomy required for extracorporeal anastomosis; however, with the development of anastomotic methods and surgical instruments, complete intracorporeal anastomosis became possible, and the trend of choice among surgeons shifted from LADG to complete LDG. As in B‐I anastomosis, many surgeons began applying complete intracorporeal delta‐shaped anastomosis (DA) in the procedure of gastroduodenal anastomosis, which was first reported by Kanaya et al. in 2002 [4], instead of the conventional anastomosis which incorporated a circular stapler (CS) inserted through a small abdominal incision.

The differences among B‐I, B‐II, and R‐Y reconstructions have been discussed in several studies [5, 6, 7, 8, 9, 10, 11, 12], for example, in terms of postoperative adverse events and endoscopic findings, which mainly focused on remnant gastritis evaluated by the residual food, gastritis, and bile‐reflux classification (RGB classification, Table 1 [13]). Although B‐I reconstruction has been reported to cause severe gastritis more frequently than other methods [5, 6, 7, 9, 10, 12], it is preferred in many institutions because it requires only one anastomosis and mostly retains preoperative physiological similarity in the digestive pathway. However, although several studies have attempted to clarify the differences between the DA and CS groups in surgical and short‐term postoperative outcomes [14, 15, 16, 17, 18, 19], nutritional effects, and changes in body composition [7, 20] or prognostic outcomes [21, 22], few studies have adequately compared the physiological status of the remnant stomach despite important differences between the anastomotic techniques. In this study, we compared not only surgical outcomes of DA and CS, but also postoperative conditions of the remnant stomach evaluated by endoscopy, with emphasis on remnant gastritis, reflux esophagitis, and hiatal hernia. We also examined whether the detected differences between the two techniques affect the degree of postoperative weight loss.

**TABLE 1:** RGB classification.

Residual food	Grade 0	No residual food.
	Grade 1	A small amount of residual food.
	Grade 2	A moderate amount of residual food, but possible to observe the entire body of the remnant stomach by body rolling.
	Grade 3	A moderate amount of residual food, which hinders observation of the entire surface.
	Grade 4	A great amount of residual food, for which endoscopic observation is impossible.
Gastritis (Degree)	Grade 0	Normal mucosa.
	Grade 1	Mild redness.
	Grade 2	Intermediate grade between Grade 1 and Grade 3.
	Grade 3	Severe redness.
	Grade 4	Apparent erosion.
Extent of gastritis	Grade 0	No gastritis.
	Grade 1	Limited to the anastomosis.
	Grade 2	Intermediate area between Grade 1 and Grade 3.
	Grade 3	Whole remnant gastritis.
Bile reflux	Grade 0	Absence of bile.
	Grade 1	Presence of bile.

## 2 Patients and Methods

### 2.1 Patients

**FIGURE 1:**
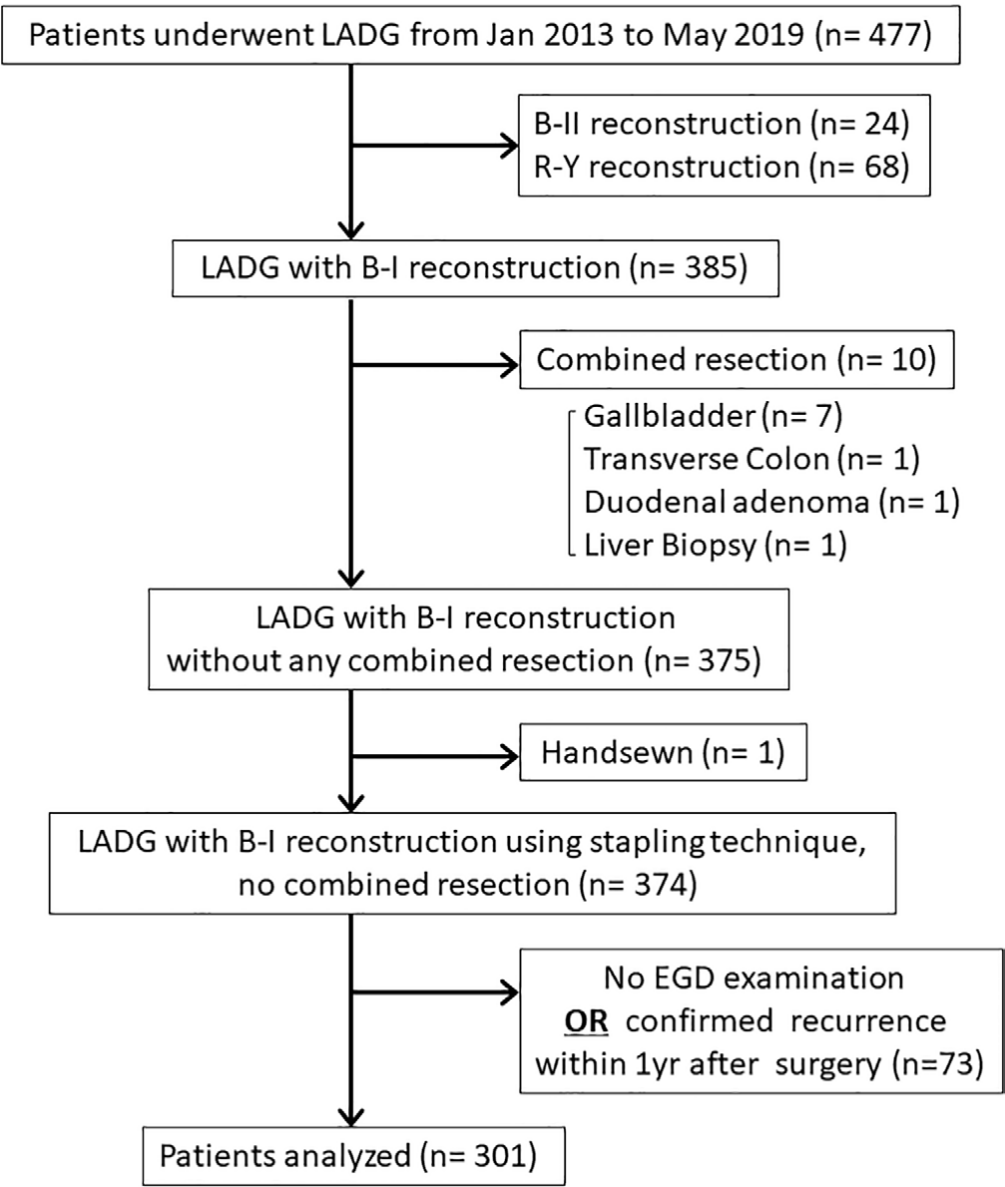
Consort diagram. B‐I, Billroth I; B‐II, Billroth II; EGD, esophagogastroduodenoscopy; LADG, laparoscopy‐assisted distal gastrectomy; R‐Y, Roux‐en‐Y.

From January 2013 to May 2019, 477 consecutive patients underwent LADG in the Department of Upper Gastrointestinal Surgery at Saitama Medical University International Medical Center. Of these, 385 underwent B‐I reconstruction, but 10 patients who underwent combined resection of other organs and 1 patient who underwent hand‐sewn anastomosis were excluded, as were 73 patients who did not undergo endoscopy between 6 and 18 months postoperatively as a first‐year checkup in the absence of recurrence. Thus, 301 patients were included in this study (Figure 1). This study was approved by the Ethics Committee of the International Medical Center of Saitama Medical University (registration number:19–226).

### 2.2 Surgery

In our department, LADG was mainly administered to patients with gastric cancer of cT3N1 or less and a tumor diameter of 5 cm or less. Gastric resection and lymph node dissection were performed with six trocars placed at the umbilicus, the upper lateral abdomens, and both the hypochondrium and the epigastric region. The procedures were well‐standardized and performed following the treatment guidelines of the Japanese Gastric Cancer Association (JGCA) [23, 24, 25] In all cases throughout the study period. However, since April 2016, the anastomotic technique has gradually shifted from extracorporeal anastomosis using a circular stapler (CS) to intracorporeal DA, which is now performed in almost all cases of B‐I reconstruction.

**FIGURE 2:**
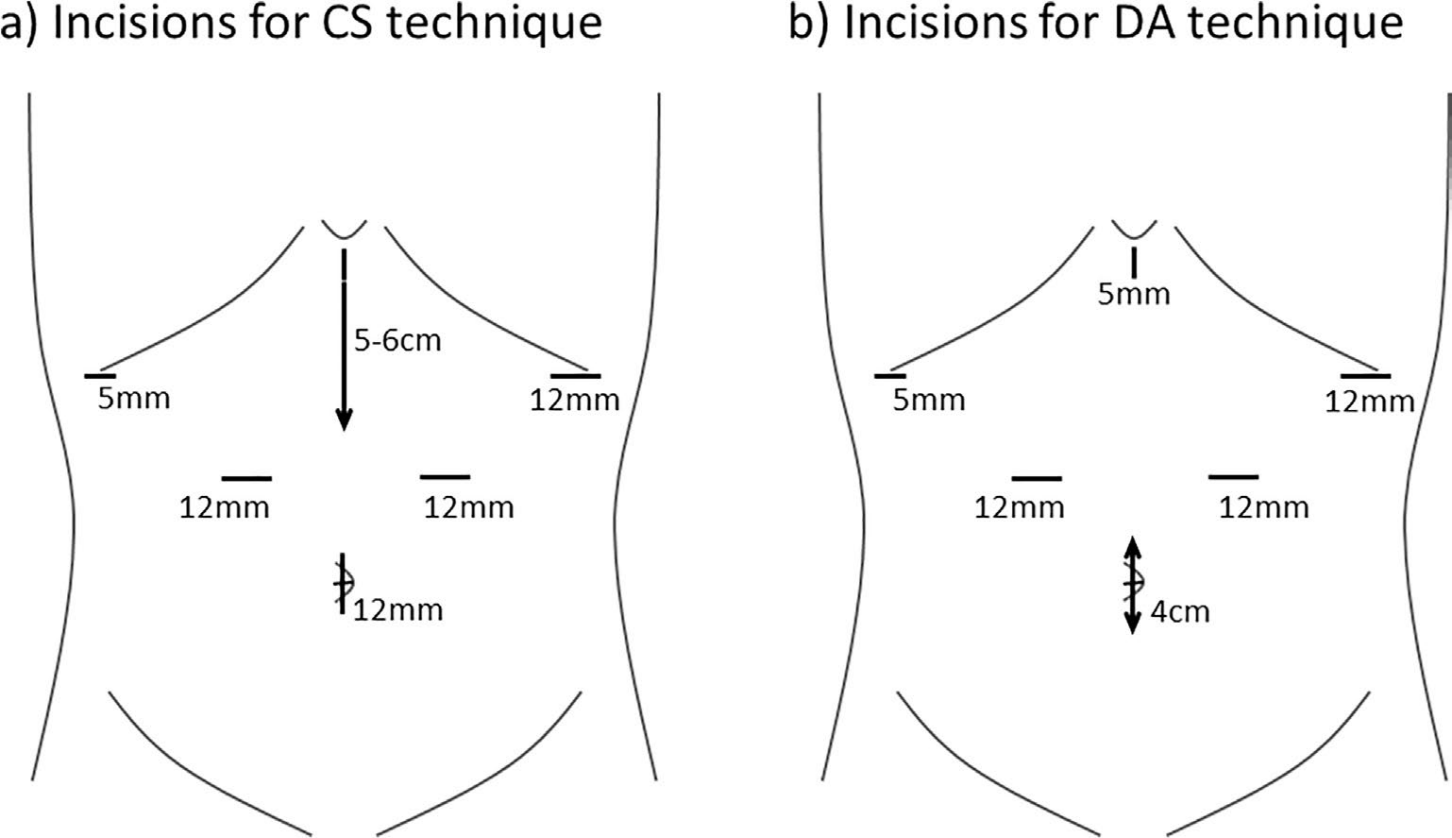
Surgical incisions for each procedure. (a) CS technique. A 5–6 cm long incision was placed in the upper abdomen caudal to the epigastric port. (b) DA technique. The umbilical port incision was extended 4 cm to remove the resected stomach. CS, circular stapled; DA, delta‐shaped anastomosis; LADG, Laparoscopy‐assisted distal gastrectomy.

In the conventional extracorporeal CS anastomosis, a small incision of approximately 5–6 cm was placed caudal to the epigastric port, and a 25‐mm circular stapler was used through the incision to anastomose the posterior wall of the remnant stomach and duodenal stump. In contrast, in the DA technique, the resected stomach was removed through an umbilical port incision extending to 4 cm. After small entry holes were made at the end of the gastric curvature of the remnant stomach and duodenal end, their posterior walls were anastomosed laterally with a 45‐mm linear stapler inserted through each hole, and the common entry hole was closed with a 60‐mm linear stapler. The placement of surgical incisions in each procedure is shown in Figure 2. Neither Kocher's mobilization nor routine crural repair was performed before the anastomosis during the surgeries.

All surgeries were conducted by surgical teams that included at least one supervising endoscopic surgeon certified by the Japanese Society of Endoscopic Surgery, either as the operator or the first assistant.

### 2.3 Clinical, Surgical, and Pathological Data Collection

Patients' clinical data, including sex, age, preoperative body mass index (BMI), and information on postoperative drug use including adjuvant chemotherapies were collected retrospectively from their medical records. Surgical information, including the extent of lymph node dissection (D1+ or less and D2 dissection), operative time, intraoperative blood loss, and postoperative adverse events of Grade 2 or higher in the Clavien‐Dindo classification [26] was also collected. Data on remnant stomach size were not recorded routinely and could not be collected. Pathological stages were determined based on the 15th Japanese Classification of Gastric Carcinoma from the JGCA [27].

### 2.4 Endoscopic Assessments

All patients included in the study underwent endoscopic examination preoperatively and in the first year postoperatively, which occurred between 6 and 18 months after surgery. The duration between the surgery and the postoperative examination was also recorded in each case.

**FIGURE 3:**
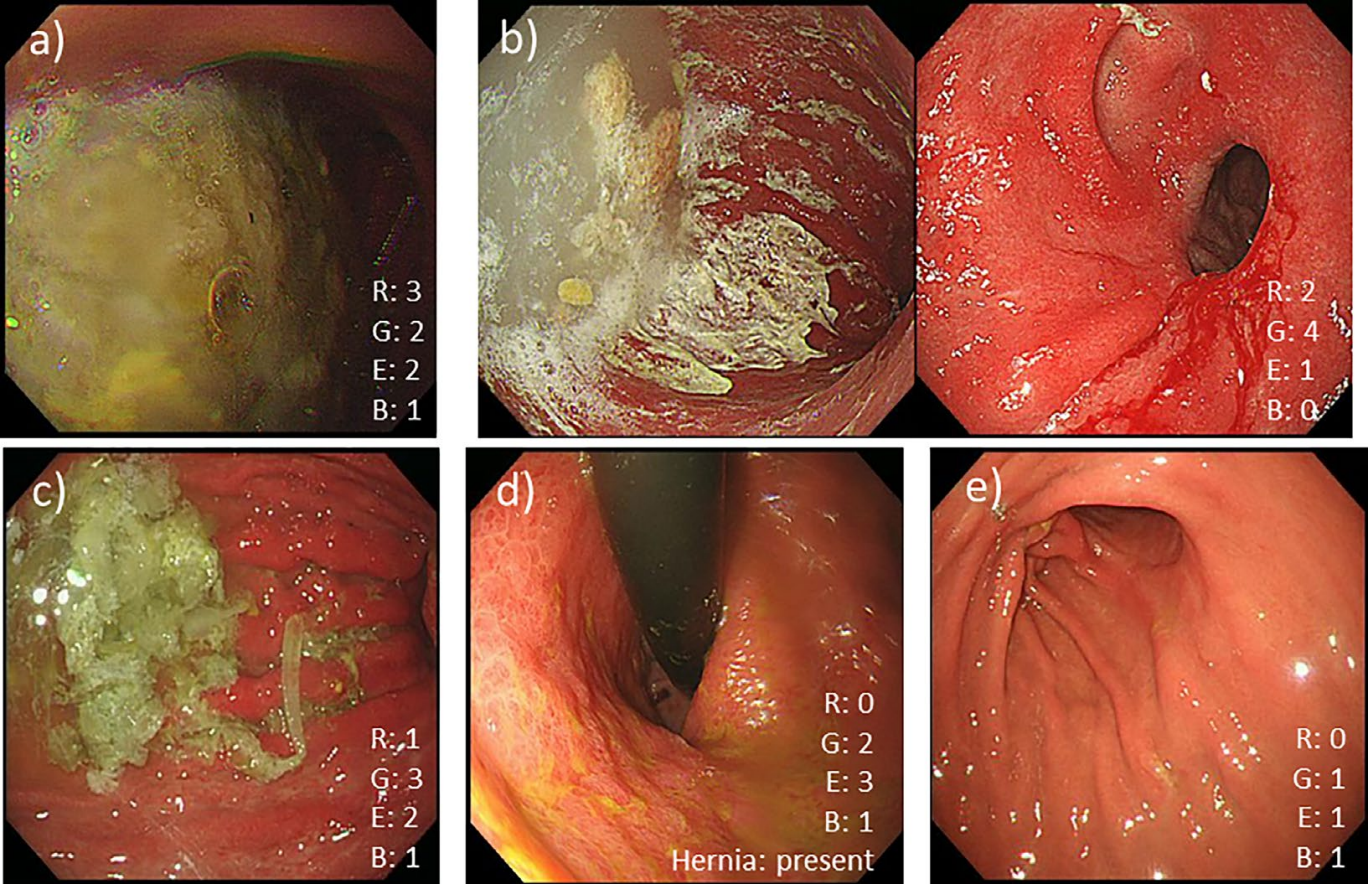
The representative endoscopic images of our cases. The RGB classification status was shown in each image. (a) A large quantity of residual food; R3. (b) Obvious erosion was limited to the anastomosis; G4E1. (c) Severe gastritis reaching up to the middle gastric body; G3E2. (d) Intermediate gastritis was seen in the whole stomach; G2E3. (e) Mild gastritis was limited to the anastomosis; G1E1.

The postoperative condition of the remnant stomach was evaluated retrospectively using the RGB classification proposed by Kubo et al. [13]. Representative images from our cases are shown in Figure 3. This classification focuses on four factors of the remaining stomach after partial gastrectomy: the amount of residual food (residue), degree of gastritis (gastritis), the extent of gastritis (extent), and presence of bile reflux (bile reflux), with high scores indicating severe cases.

The presence or absence of reflux esophagitis and hiatal hernia was retrospectively confirmed using preoperative and postoperative endoscopic images. The grade of reflux esophagitis was determined using the Los Angeles classification [28], and postoperative exacerbation of esophagitis was defined as the transition from preoperative grade M or lower to postoperative grade A or higher. Postoperative exacerbation of the hiatal hernia was defined as a newly detected postoperative hiatal hernia that was not present preoperatively. Since the *Helicobacter Pylori* infection status was unknown in many patients, the presence or absence of atrophic gastritis was instead confirmed by preoperative endoscopic images and classified into none, closed type, and open type according to the Kimura‐Takemoto classification [29]. The administration status of anti‐reflux and motility agents at the time of postoperative endoscopy was also extracted retrospectively from the medical records.

### 2.5 Postoperative Body Weight Changes

The patients' preoperative and first‐year postoperative weights were verified using the medical records. However, owing to the retrospective nature of this study, some patients had missing postoperative weight data, and these patients were excluded from the analysis of postoperative weight loss to examine the impact of clinical, surgical, pathological, and endoscopic factors.

### 2.6 Statistical Analysis

Statistical analysis was performed using JMP 14 software (SAS Institute Inc., Cary, NC, USA). For univariate analysis showing differences between groups, Fisher's exact tests were applied for categorical variables and the Wilcoxon rank‐sum test was applied for continuous variables. Multivariate logistic regression analyses of categorical outcomes were performed using factors with *p* values less than 0.3 in the univariate analyses. Two‐sided *p* values of less than 0.05 were considered significant.

## 3 Results

### 3.1 Patients' Characteristics and Surgical Outcomes

Of the 301 patients, 150 underwent the CS technique and 151 underwent the DA technique. The characteristics of patients in each group and their surgical outcomes are shown in Table 2. In the univariate analysis, there were no significant differences in background factors except for preoperative BMI (CS 21.9 [20.2, 23.2]: DA 22.6 [20.8, 25.3], *p* = 0.0182) which was very small. We did not intend to select the anastomosis method according to the patient's BMI, and the methods were determined mostly by when the procedure was performed at our institution. There was also no difference in the extent of lymph node dissection or the pathological stage. The operative time and intraoperative blood loss were also not significantly different between the groups, and multivariate analyses for these factors showed that the CS technique significantly increased intraoperative blood loss but not the operative time (Table S1).

**TABLE 2:** Patient characteristics and surgical outcome with univariate analyses.

		All	Circular stapler			Delta‐shaped anastomosis			*p*
Number of patients		301	150			151			
Age		69	68	[62, 74]		70	[63, 74]		0.6816
Sex	Male	207	100		66.7%	107		70.9%	0.4572
	Female	94	50		33.3%	44		29.1%	
BMI		22.2	21.9	[20.2, 23.7]		22.6	[20.8, 25.3]		0.0182*
Lymphadenectomy	≤ D1+	241	116		77.3%	125		82.8%	0.2514
	D2	60	34		22.7%	26		17.2%	
pStage(JGCA 15th)	IAB	249	121		80.7%	128		84.8%	0.3642
	IIAB/IIIABC/IV	52	29		19.3%	23		15.2%	
Operative time (min.)		244	239	[213, 273]		247	[217, 284]		0.1431
Blood Loss (mL)		20	21	[5, 50]		10	[5, 30]		0.1045
Adverse events (*n*=, %)	Any	33	20	13.3%		13	8.6%		0.2020
(≥ CD Grade2)	Anast. Leakage	2	1	0.7%		1	0.7%		1.0000
	POPF	1	0	0.0%		1	0.7%		1.0000
	Abscess	6	0	0.0%		6	4.0%		0.0297*
	Respiratory	7	7	4.7%		0	0.0%		0.0071*
	DGE	6	4	2.7%		2	1.3%		0.4476
	Ileus	2	2	1.3%		0	0.0%		0.2475

*Note:* Median [25%tile, 75%tile] is shown for continuous variables.

Abbreviations: BMI, body mass index; CD, clavien‐dindo classification; DGE, delayed gastric emptying; JGCA, Japanese Gastric Cancer Association; POPF, postoperative pancreatic fistula; pStage, pathological stage.

* *p* < 0.05.

Among the postoperative complications of Grade 2 or higher in the Clavien‐Dindo classification listed in the table, the incidence rate of abdominal abscess was significantly higher in the DA group (CS 0.0%: DA 4.0%, *p* = 0.0297). However, abscesses occurred more frequently in the early years of the introduction of the DA technique in our department (6.5% in the first 76 cases) and decreased after the technique was standardized (1.3% in the later 75 cases). In comparison, the incidence rate of respiratory problems such as pneumonia was significantly higher in the CS group (CS 4.7%: DA 0.0%, *p* = 0.0071).

### 3.2 Endoscopic Findings

The pre‐ and postoperative endoscopic findings are shown in Table 3, along with information on the administration of anti‐reflux and motility agents. The results of the univariate analysis are also shown. The postoperative observational period was comparable between the CS and DA groups (CS 12.0 months [11.7, 12.3]: DA 11.9 months [11.5, 12.4]; *p* = 0.5196).

**TABLE 3:** Preoperative and postoperative endoscopic findings and administration of relevant agents.

		All	Circular stapler			Delta‐shaped			*p*
Number of patients		301	150			151			
Obs. period (mths)		11.9	12	[11.7, 12.3]		11.9	[11.5, 12.4]		0.5196
Residue	Grade 0/1	194	90	74/16	60.0%	104	84/20	68.9%	0.1184
	Grade 2/3/4	107	60	13/44/3	40.0%	47	12/34/1	31.1%	
Gastritis	Grade 0/1	224	126	65/61	84.0%	98	36/62	64.9%	0.0002*
	Grade 2/3/4	77	24	22/1/1	16.0%	53	46/7/0	35.1%	
Extent	Grade 0/1	209	109	65/44	72.7%	100	36/64	66.2%	0.2604
	Grade 2/3	92	41	39/2	27.3%	51	44/7	33.8%	
Bile reflux	Grade 0	94	53		35.3%	41		27.2%	0.1368
	Grade 1	207	97		64.7%	110		72.8%	
Hiatal hernia(*n*=, %)	preoperative	95	51	34.0%		44	29.1%		0.3871
	postoperative	169	87	58.0%		82	54.3%		0.5620
	exacerbation	85	42	28.0%		43	28.5%		1.0000
GERD ≥ GradeA (*n*=, %)	preoperative	11	3	2.0%		8	5.3%		0.2179
	postoperative	18	4	2.7%		14	9.3%		0.0261*
	exacerbation	14	3	2.0%		11	7.3%		0.0517**
stricture dilation (*n*=, %)		0	0	0.0%		0	0.0%		1.0000
Anti‐reflux drug use (*n*=, %)		29	20	13.3%		9	6.0%		0.0329*
motility drug use (*n*=, %)		21	13	8.7%		8	5.3%		0.2680
Adjuvant CTx	yes	35	21		14.0%	14		9.3%	0.2134
Atrophy	None/Closed	106	56		37.3%	50		33.1%	0.4705
	Open	195	94		62.7%	101		66.9%	

*Note:* Median [25%tile, 75%tile] is shown for continuous variables.

Abrreviations: JGCA, Japanese Gastric Cancer Association; Obs, observational period; mths months, anastomosis; RE, reflux esophagitis.

* *p* < 0.05;

** *p* < 0.1.

Postoperative findings were divided into two subgroups according to the level of RGB classification Grade 1 or lower and Grade 2 or higher. This was because we usually consider additional drug administration in patients with higher grades at these cutoffs. The amount of residual food was greater in the CS group, which was not statistically significant (*p* = 0.1184). Similarly analyzed, the degree score of remnant gastritis was significantly higher in the DA group (Grade 2 or higher rates were CS 16.0%: DA 35.1%, *p* = 0.0002), and the extent score was also higher in the DA group although it was not significant (Grade 2 or higher rates were CS 27.3%: DA 33.8%, *p* = 0.2604). Bile reflux was more common in the DA group, but this was not significantly different (Grade 1 rates were CS 64.7%: DA 72.8%, *p* = 0.1368).

The preoperative and postoperative prevalence rates of hiatal hernia did not differ between the two groups. Still, in the DA group, postoperative reflux esophagitis was significantly more frequent (CS 2.7%: DA 9.3%, *p* = 0.0261) and the exacerbation rate tended to be higher (CS 2.0%: DA 7.3%, *p* = 0.0513). The administration status of anti‐reflux and motility agents and the extent of preoperative gastric mucosal atrophy were not significantly different between the two groups.

For the degree of remnant gastritis, postoperative reflux esophagitis, and exacerbation of reflux esophagitis, which seemed to differ depending on the anastomosis technique, we performed multivariate analyses that included clinicopathological factors as variables (Table 4). The DA technique was a significant risk factor for all the mentioned endoscopic findings (OR [95% CI] was 2.737 [1.566–4783] for gastritis, 3.533 [1.101–11.34] for postoperative reflux esophagitis, and 3.749 [1.021–13.76] for exacerbated reflux esophagitis). Other significant risk factors were male sex (OR [95% CI] was 2.081 [1.091–3.968]) for remnant gastritis, less preoperative gastric mucosal atrophy (OR [95% CI] was 2.946 [1.043–8.316]) for postoperative reflux esophagitis. On the other hand, no significant risk factors other than anastomotic technique were found for exacerbation of reflux esophagitis.

**TABLE 4:** Univariate and multivariate analyses of endoscopic findings.

(a) The degree of gastritis									
		Gastritis				*p*		OR	[95% CI]
		Grade0–1		Grade2–4		Univariate	Multivariate		
Number of patients		224		77					
Age		68	[61, 74]	70	[65, 74]	0.1610	0.3547	—	
Sex	Male	145		62		0.0103*	0.0210*	2.081	[1.091, 3.968]
	Female	79		15				1	
BMI		22.4	[20.4, 24.5]	22.2	[20.6, 24.4]	0.9768	—		
Lymphadenectomy	≤ D1+	179		62		1.0000	—		
	D2	45		15					
Anastomosis	Circular stapler	126		24		0.0002*	0.0003*	1	
	Delta‐shaped	98		53				2.737	[1.566, 4.783]
pStage (JGCA, 15th)	IAB	184		65		0.7288	—		
	IIAB/IIIABC/IV	40		12					
Adverse events ≥ C‐D Grade 2 (*n*=, %)		25	11.2%	8	10.4%	1.0000	—		
Anti‐reflux drug use (*n*=, %)		23	10.3%	6	7.8%	0.6566	—		
motility drug use (*n*=, %)		16	7.1%	5	6.5%	1.0000	—		
Adjuvant Chemotherapy (*n*=, %)		29	13.0%	6	7.8%	0.3030			
Atrophy	None/Closed	80		26		0.7839	—		
	Open	144		51					
Obs. Period (mths)		11.9	[11.6, 12.4]	11.9	[11.4, 12.3]	0.0965	0.1959	—	

(b) Postoperative reflux esophagitis									
		Postoperative RE				*p*		OR	[95% CI]
		No		Yes		Univariate	Multivariate		
Number of patients		283		18					
Age		69	[63, 74]	71	[61, 78]	0.1947	0.1628	—	
Sex	Male	191		16		0.0675*	0.0759	—	
	Female	92		2					
BMI		22.3	[20.5, 24.5]	22	[19.1, 24.0]	0.7491	—		
Lymphadenectomy	≤D1+	224		17		0.1384	0.1766	—	
	D2	59		1					
Anastomosis	Circular stapler	146		4		0.0261*	0.0338*	1	
	Delta‐shaped	137		14				3.533	[1.101, 11.34]
pStage (JGCA, 15th)	IAB	233		16		0.7480	—		
	IIAB/IIIABC/IV	50		2					
Adverse events ≥ C‐D Grade2 (*n*=, %)		33	11.7%	0	0.0%	0.2366	0.9978	—	
Anti‐reflux drug use (*n*=, %)		27	9.5%	2	11.1%	0.6877	—		
motility drug use (*n*=, %)		21	7.4%	0	0.0%	0.6242	—		
Adjuvant chemotherapy (*n*=, %)		34	12.0%	1	5.6%	0.7052	—		
Atrophy	None/Closed	97		9		0.2058	0.0412*	2.946	[1.043, 8.316]
	Open	186		9				1	
Obs. Period (mths)		11.9	[11.6, 12.3]	11.9	[11.5, 12.4]	0.7213	—		

(c) Exacerbated reflux esophagitis									
		Exacerbated RE				*p*		OR	[95% CI]
		No		Yes		Univariate	Multivariate		
Number of patients		287		14					
Age		69	[63, 74]	70	[59, 78]	0.5309	—		
Sex	Male	195		12		0.2390	0.2008	—	
	Female	92		2					
BMI		22.2	[20.5, 24.5]	22.3	[19.2, 24.0]	0.7725	—		
Lymphadenectomy	≤ D1+	228		13		0.3161	—		
	D2	59		1					
Anastomosis	Circular Stapler	147		3		0.0517**	0.0464*	1	
	Delta‐shaped	140		11				3.749	[1.021, 13.76]
pStage (JGCA, 15th)	IAB	236		13		0.4772	—		
	IIAB/IIIABC/IV	51		1					
Adverse events ≥ C‐D Grade2 (*n*=, %)		33	11.5%	0	0.0%	0.3782	—		
Anti‐reflux drug use (*n*=, %)		27	9.4%	2	14.3%	0.6329	—		
motility drug use (*n*=, %)		21	7.3%	0	0.0%	0.6092	—		
Adjuvant Chemotherapy (*n*=, %)		34	11.9%	1	7.1%	1.0000	—		
Atrophy	None/Closed	100		6		0.5732	—		
	Open	187		8					
Obs. Period (mths)		11.9	[11.6, 12.3]	12.1	[11.5, 12.4]	0.7894	—		

*Note:* Median [25%tile, 75%tile] is shown for continuous variables.

Abbreviations: BMI, body mass index; CD, clavien‐dindo classification; CI, confidence interval; Obs, observational, months; OR, odds ratio; RE, reflux esophagitis.

* *p* < 0.05;

** *p* < 0.1.

### 3.3 The Relationship Between Endoscopic Findings and Postoperative Weight Loss

To determine whether these significant differences in endoscopic findings and the anastomosis technique itself influence the degree of postoperative weight loss, we also conducted univariate and multivariate analysis for severe weight loss, defined as a loss of over 10% from the preoperative weight, using clinical, pathological, and endoscopic factors as variables. Since the body weight in the first postoperative year was not available for 60 patients, a total of 241 patients were analyzed, and the median weight loss rate of these patients was 8.5%. Other than the patient's preoperative BMI, the only significant endoscopic risk factor for severe weight loss was the presence of extensive remnant gastritis of Grade 2 or higher in the RGB classification (OR [95% CI] was 1.926 [1.082–3.426]), but not the degree of gastritis, exacerbation of reflux esophagitis, or anastomosis technique (Table 5).

**TABLE 5:** Univariate and multivariate analyses of postoperative weight loss severity.

		All			BW decrease						*p*		OR	[95% CI]
					Mild (≤ 10%)			Severe (> 10%)			Univariate	Multivariate		
number of patients	All	301												
	data unavailable	60												
	Analyzed	241			134			107						
BW change %		−8.5%	[−13.1, −4.9]		−5.4%	[−7.5, −1.9]		−13.7%	[−17.1, −11.2]					
Age		69	[63, 74]		68	[61, 74]		70	[63, 74]		0.9180	—		
Sex	Male	158		65.6%	90		67.2%	68		63.6%	0.5870	—		
	Female	83		34.4%	44		32.8%	39		36.4%				
BMI		22.2	[20.5, 24.4]		22.1	[20.0, 24.0]		22.6	[21.0, 25.1]		0.0133*	0.0087*	1.122	[1.027, 1.225]
Lymphadenectomy	≤ D1+	192		79.7%	109		81.3%	83		77.6%	0.5208	—		
	D2	49		20.3%	25		18.7%	24		22.4%				
Anastomosis	Circular Staper	124		51.5%	68		50.7%	56		52.3%	0.8969	—		
	Delta‐shaped	117		48.5%	66		49.3%	51		47.7%				
pStage	IAB	203		84.2%	113		84.3%	90		84.1%	1.0000	—		
	IIAB/IIIABC/IV	38		15.8%	21		15.7%	17		15.9%				
Adverse events ≥ C‐D Grade2 (*n*=, %)		25	10.4%		9	6.7%		16	15.0%		0.0541**	0.0905**		
Residue	Grade 0–1	154		63.9%	83		61.9%	71		66.4%	0.5022	—		
	Grade 2–4	87		36.1%	51		38.1%	36		33.6%				
Gastritis	Grade 0–1	183		75.9%	102		76.1%	81		75.7%	1.0000	—		
	Grade 2–4	58		24.1%	32		23.9%	26		24.3%				
Extent	Grade 0–1	168		69.7%	101		75.4%	67		62.6%	0.0352*	0.0258*	1	
	Grade 2–3	73		30.3%	33		24.6%	40		37.4%			1.926	[1.082, 3.426]
Bile reflux	Grade 0	77		32.0%	46		34.3%	31		29.0%	0.4062	—		
	Grade 1	164		68.0%	88		65.7%	76		71.0%				
exacerbated Hiatal Hernia (*n*=, %)		71	29.5%		41	30.6%		30	28.0%		0.6729	—		
exacerbated RE(*n*=, %)		9	3.7%		6	4.5%		3	2.8%		0.7348	—		
Anti‐reflux drug use (*n*=, %)		23	9.5%		12	9.0%		11	10.3%		0.8263	—		
motility drug use (*n*=, %)		17	7.1%		7	5.2%		10	9.4%		0.3113	—		
Adjuvant CTx	yes	26		10.8%	12		9.0%	14		13.0%	0.4037	—		
	no	215		89.2%	122		91.0%	93		87.0%				
Atrophy	None/Closed	81		33.6%	47		35.1%	34		31.8%	0.6807	—		
	Open	160		66.4%	87		64.9%	73		68.2%				
Obs. Period (mths)		11.9	[11.6, 12.3]		11.9	[11.6, 12.3]		12	[11.6, 12.5]		0.1073	0.1262	—	

*Note:* Median [25%tile, 75%tile] is shown for continuous variables.

Abbreviations: BMI, body mass index; BW, body weight; CD, clavien‐dindo classification; CI, confidence interval, CTx, chemotherapy; Obs, Observational, months; OR, odds ratio; RE, reflux esophagitis.

* *p* < 0.05.

** *p* < 0.1.

## 4 Discussion

Although DA is increasingly preferred over CS in many institutions because of the simplicity of the anastomosis technique, there are not many reports on the characteristics of the intraoperative and postoperative outcomes of each anastomosis technique. We therefore conducted this study to understand the physiological differences between the two anastomoses by detecting differences in postoperative endoscopic findings.

Comparing the surgical outcomes of the DA and CS groups, there was no significant difference in the median operative time. However, the intraoperative blood loss tended to increase in the CS group. This may be due to the upper abdominal incision and temporary release from abdominal air ventilation in the CS technique, which may promote intraoperative micro‐bleeding. Therefore, the DA method seems advantageous in reducing intraoperative bleeding, although the difference may be small.

The total incidence rates of surgical adverse events of Grade 2 or higher in the Clavien‐Dindo classification were not significantly different between the two anastomotic techniques consistent with previous reports [14, 15, 16, 17, 21]. Among them, postoperative pneumonia was more common in the CS group due to the upper abdominal incision as well reported in many studies. On the other hand, postoperative abdominal abscesses were more common after the DA anastomosis, possibly due to the higher risk of gastric and duodenal contents leakage during the complete intracorporeal procedure. However, since the abscesses occurred mainly in the early period after the introduction of the DA method, the incidence is expected to decrease as surgeons become more proficient.

Next, we compared the postoperative endoscopic findings between the DA and CS groups. The degree of remnant gastritis and the occurrence rates of postoperative and exacerbated reflux esophagitis were significantly higher in the DA group than in the CS group, even in the multivariate analysis. The amount of residual food was higher in the CS group, and the incidence of bile reflux was higher in the DA group, although these were not statistically significant.

In the previous study, Lee et al. discussed the postoperative endoscopic findings after each anastomotic technique and showed that bile reflux was more severe in the DA group throughout the first 5 postoperative periods than in the CS group. Their findings were consistent with our results, but the severity and extent of gastritis were worse in the CS group than in the DA group, especially in the first year after surgery [21]. Their results of remnant gastritis were opposite to ours, but since the primary mechanism of remnant gastritis is thought to be the result of chronic bile reflux [30, 31], gastritis can be more prevalent in the DA group, which was more associated with bile reflux.

Additionally, the DA technique was associated with a higher incidence of postoperative reflux esophagitis and exacerbation of esophagitis; however, this has not been discussed much in previous reports. In this study, the DA technique was the only significant risk factor for esophagitis exacerbation, but less preoperative gastric atrophy was also associated with postoperative reflux esophagitis. This is consistent with the previous report which shows that the mixture of bile juice and gastric acid can cause more severe mucosal damage on the esophagogastric junction after upper gastrointestinal surgery [32, 33].

All of the significant endoscopic differences identified in this study appear to be associated with a higher incidence of biliary reflux. One possible reason for these findings may be the wider lumen of the anastomosis in the DA technique, as mathematically estimated in the study by Park et al. [20]. This can also be the explanation for the more residual food in the CS groups. Another reason may be that the anastomosis in the CS method is usually performed with an end‐to‐side anastomosis with the duodenum dorsal to the residual stomach, whereas in the DA method, the anastomosis is almost straight with a side‐to‐side anastomosis.

Since postoperative discomfort due to remnant gastritis or reflux esophagitis and the anastomotic technique may affect patients' eating habits and nutritional status, we hypothesized that these factors would also affect the degree of postoperative weight loss. Although the postoperative body weight data were unavailable for some patients, the median postoperative weight loss rate was 8.5% in this study which was nearly identical to the rates reported in other studies [7, 20]. Our results showed that the anastomosis technique, the degree of remnant gastritis, or the exacerbation of reflux esophagitis did not make a significant difference in postoperative body weight, but that the extent of gastritis was an important factor for severe weight loss. One possible reason for this result is that extensive chronic remnant gastritis reaching the original fundus region may strongly reduce ghrelin levels and intensify weight loss, and further studies are required to confirm this point.

In summary, the intensity of remnant gastritis and the incidence of postoperative erosive reflux esophagitis were higher with the DA technique than with the CS technique. However, these endoscopic findings did not affect postoperative weight loss, and the only endoscopically relevant factor was the extent of residual gastritis, which did not differ significantly between the two anastomotic techniques. Since the DA method seems to have the advantage of reducing intraoperative bleeding and postoperative pneumonia, it would be reasonable to choose the DA method over the CS method as long as the possibility of an increased risk of residual gastritis and postoperative reflux esophagitis is fully understood.

This study has several limitations. First, the two anastomosis methods were performed at different times, which may have led to minor differences in surgical techniques and perioperative care. Second, data on residual stomach size, which could influence postoperative endoscopic outcomes, were unavailable [34]. Third, due to the study's retrospective design, we could not assess patients' subjective symptoms using tools like the Post‐Gastrectomy Syndrome Assessment Scale Questionnaire. Fourth, we could not fully account for other hospitals' anti‐reflux and motility drug prescriptions, though these drugs did not significantly affect our findings. Fifth, missing postoperative weight data limited the multivariate analysis for severe weight loss, but the results still provide valuable insights into weight loss and anastomotic techniques. Sixth, differences in follow‐up durations prevented a prognostic comparison between anastomosis methods. Seventh, as the study included only Japanese patients, the results might differ in other ethnic groups due to variations in preoperative gastric conditions, such as *Helicobacter pylori* infection. Lastly, repeated statistical analyses might have introduced some multiplicity issues, though they were necessary for evaluating individual endoscopic findings.

## 5 Conclusion

In this study, the DA technique had higher incidences of remnant gastritis or exacerbated reflux esophagitis, while the CS technique tended to have more food residue. These differences in endoscopic findings by anastomotic technique were not directly associated with severe weight loss. However, the extent of remnant gastritis can affect the patient's postoperative weight changes and should be carefully monitored during outpatient follow‐up.

## Author Contributions

All authors made significant contributions to the design and analysis of this study and the drafting and evaluation of the manuscript. All authors acknowledged submission of the final manuscript and agreed to be accountable for this study.

## Ethics Statement

All procedures were performed according to the ethical standards of the responsible committee on human experimentation (institutional and national) and in accordance with the Helsinki Declaration of 1964 and later versions.

## Consent

Informed consent to be included in the study or equivalent was obtained from all the patients.

## Conflicts of Interest

The authors declare no conflicts of interest.

## Supporting information


**Table S1** Multivariate analyses for (a) operative time and (b) intraoperative blood loss.

## Data Availability

The data that support the findings of this study are available on request from the corresponding author. The data are not publicly available due to privacy or ethical restrictions.
