# Supplementary material for: Comparisons in Postoperative Endoscopic Findings and Postoperative Weight Change Between Delta‐Shaped Anastomosis and Circular‐Stapled Anastomosis in Laparoscopy‐Assisted Distal Gastrectomy With B‐I Reconstruction
Source: Asian J Endosc Surg. 2025 Jan 23;18(1):e70023. doi: 10.1111/ases.70023 (PMC11757014; doi:10.1111/ases.70023)
Supplement: Supplementary file 1 — Table S1 Multivariate analyses for (a) operative time and (b) intraoperative blood loss. [file ASES-18-e70023-s001.docx]

Supplementary Table. Multivariate analyses for a) operative time and b) intraoperative blood loss

| **a)** |  |  |  |  |  |  |  |
| --- | --- | --- | --- | --- | --- | --- | --- |
|  |  | Operative time | | | | | |
|  |  | B(Coefficient) | SE | [95% CI] | P value | *Beta* | Interpretation |
|  | Constant | 203.97 | 26.032 |  |  |  |  |
| Age |  | -0.0655 | 0.2379 | [-0.5338, 0.4027] | 0.7831 | -0.0151 | - |
| Sex | Female / Male | -11.7378 | 2.8284 | [-17.3043, -6.1713] | <0.0001* | -0.2394 | Longer with Male |
| BMI |  | 2.2984 | 0.8477 | [0.6300, 3.9668] | 0.0071* | 0.1565 | Longer with obesity |
| Lymphadenectomy | =<D1+ / D2 | -8.3389 | 3.3442 | [-14.9206, -1.7571] | 0.0132* | -0.1466 | Longer with D2 |
| Anastomosis | CS / DA | -3.1679 | 2.4931 | [-8.0747, 1.7387] | 0.2049 | -0.0697 | - |
| pStage (JGCA) | pStageI / pStageII-IV | -2.5373 | 3.5392 | [-9.5027, 4.4281] | 0.4740 | -0.0422 | - |
|  |  |  |  |  |  |  |  |
| **b)** |  |  |  |  |  |  |  |
|  |  | Intraoperative Blood Loss | | | | | |
|  |  | B(Coefficient) | SE | [95% CI] | P value | *Beta* | Interpretation |
|  | constant | -6.4196 | 30.637 |  |  |  |  |
| Age |  | -0.3511 | 0.2800 | [-0.9023, 0.2000] | 0.2109 | -0.0714 | - |
| Sex | Female / Male | -5.6931 | 3.3287 | [-12.2443, 0.8581] | 0.0883 | -0.1023 | - |
| BMI |  | 2.8125 | 0.9977 | [0.8489, 4.7760] | 0.0051* | 0.1687 | More with obesity |
| Lymphadenectomy | =<D1+ / D2 | -4.1419 | 3.9359 | [-11.8880, 3.6041] | 0.2935 | -0.0641 | - |
| Anastomosis | CS / DA | 7.9111 | 2.9342 | [2.1364, 13.6858] | 0.0074* | 0.1533 | More with CS |
| pStage (JGCA) | pStageI / pStageII-IV | -0.5702 | 4.1653 | [-8.7678, 7.6274] | 0.8912 | -0.0083 | - |

The least squares method was used for this analysis. BMI body mass index, JGCA Japanese Gastric Cancer Association, SE Standardized error, Beta Standardized coefficient. * p<0.05
